# Aquaporin‐3 and aquaporin‐5 impact the development of pancreatic ductal adenocarcinoma spheroids

**DOI:** 10.1002/2211-5463.70270

**Published:** 2026-05-17

**Authors:** Catarina Pimpão, Diogo M. Engrácia, Graça Soveral, Filipa Mendes

**Affiliations:** ^1^ Research Institute for Medicines (iMed.ULisboa), Faculty of Pharmacy University of Lisbon Portugal; ^2^ Department of Pharmaceutical Sciences and Medicines, Faculty of Pharmacy University of Lisbon Portugal; ^3^ C2TN ‐ Centro de Ciências e Tecnologias Nucleares Instituto Superior Técnico, Universidade de Lisboa Bobadela Portugal; ^4^ Departamento de Engenharia e Ciências Nucleares Instituto Superior Técnico, Universidade de Lisboa Bobadela Portugal

**Keywords:** aquaporins, growth, inhibitors, pancreatic ductal adenocarcinoma, spheroids

## Abstract

Pancreatic ductal adenocarcinoma (PDAC) is the seventh leading cause of cancer‐related mortality, with poor survival due to late diagnosis and ineffective therapies. Aquaporin‐3 (AQP3) and AQP5 are transmembrane proteins overexpressed in PDAC, promoting tumor progression and metastasis, representing promising therapeutic targets. Here, we investigated their involvement in PDAC spheroids' growth and morphology using two human PDAC cell lines, BxPC‐3 and MiaPaca‐2. Treatment with AQP3 inhibitors decreased spheroids' diameter, area, and viability and altered MiaPaca‐2 spheroids' circularity, suggesting reduced growth. Similarly, silencing AQP3 or AQP5 in BxPC‐3 spheroids decreased spheroids' size with a more pronounced viability reduction, indicating impaired growth and cell death. This study demonstrates, for the first time, the critical roles of AQP3 and AQP5 in PDAC spheroids' development.

## Graphical abstract

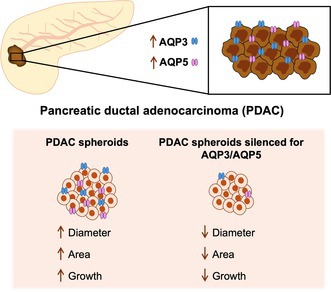

**Abbreviations**
DMEM: Dulbecco's modified Eagle's medium
ECL: Enhanced chemiluminescence
ERK: Extracellular signal‐regulated kinase
FBS: Fetal bovine serum
HPRT‐1: Hypoxantine phosphoribosyltransferase 1
KD: knockdown
MAPK: Mitogen activated protein kinase
PBS: Phosphate buffered saline
P_f_
: Water permeability
P_gly_
: Glycerol permeability
qPCR: quantitative PCR
RPMI: Roswell Park Memorial Institute
SD: Standard deviation
sgRNAs: Single guide RNAs

Pancreatic ductal adenocarcinoma (PDAC) is a highly aggressive and lethal malignancy, representing over 90% of pancreatic cancer cases and being the seventh leading cause of cancer‐related mortality [1]. The 5‐year survival rate for PDAC is less than 10%, primarily due to late diagnosis and the limited efficacy of the current treatment options [2, 3]. Although surgical resection and chemotherapy remain the standard therapeutic approaches for PDAC, their efficiency is drastically reduced in advanced stages of the disease [2, 4]. Recently, immunotherapies, such as immune checkpoint inhibitors, have emerged as promising therapeutic strategies; however, they are not able to overcome the challenging PDAC tumor microenvironment, composed of diverse cellular components and a dense extracellular matrix that greatly contributes to the resistance to therapy and immunosuppressive features [5]. Therefore, further research is needed to identify new therapeutic targets in PDAC and develop novel anticancer therapies that can impact cancer progression.

Aquaporins (AQPs) have gained attention as promising therapeutic targets in PDAC due to their significant involvement in tumor progression and metastasis [6]. AQPs are transmembrane proteins that mediate the transport of water, glycerol, H_2_O_2_, and other small solutes across cellular membranes, in response to osmotic or solute gradients [7, 8]. Thirteen AQP isoforms (AQP0–12) have been identified in humans, being differentially expressed in every tissue and cell type and playing important roles in water and energy homeostasis [9]. AQPs can be categorized into three main subgroups: classical or orthodox aquaporins (AQP0, AQP1, AQP2, AQP4, AQP5, AQP6, and AQP8), mainly selective to water; aquaglyceroporins (AQP3, AQP7, AQP9, and AQP10), that also permeate glycerol and other small solutes; and unorthodox or subcellular aquaporins (AQP11 and AQP12) with distinct evolutionary pathways, intracellular localization, and uncertain permeability features [10, 11]. An overlapping subgroup has been identified, comprising isoforms that facilitate the permeation of hydrogen peroxide (H_2_O_2_), called peroxiporins (AQP0, AQP1, AQP3, AQP5, AQP8, AQP9, and AQP11) [12, 13].

AQP3 and AQP5 are overexpressed in PDAC, playing a critical role in its progression [6]. AQP3 protein expression is significantly elevated in PDAC tumors compared to healthy tissues, being correlated with higher lymph node metastasis and invasion [14]. In addition, AQP3 was found to decrease apoptosis and promote PDAC proliferation, probably by the activation of the mTOR signaling pathway [15], while AQP5 contributes to both cancer cell migration and invasion [16]. Our research group has demonstrated that AQP3 expression levels increase from early to later stages of PDAC, whereas AQP5 expression is higher in early stages but becomes almost undetectable in advanced stages of the disease [17]. Both AQP3 and AQP5 overexpression were correlated with enhanced expression of epidermal growth factor receptor (EGFR), proliferation marker Ki‐67, cytokeratin 7, and vimentin, as well as reduced expression of E‐cadherin, indicating the contribution of AQP3 and AQP5 to epithelial‐mesenchymal transition (EMT) and tumorigenesis [17]. Additionally, we observed that AQP3‐, AQP5‐, and double‐silenced cells showed an impairment in PDAC cell migration, exhibiting morphological alterations and decreased cell–cell adhesion [18, 19]. Moreover, AQP5 silencing impacted both cell stiffness and membrane fluidity [19]. In a complementary model using HEK‐293 T cells, AQP3 and AQP5 overexpression exerted contrasting effects on cell stiffness and cell–cell adhesion, with AQP3 increasing stiffness and reducing cell–cell [20]. Overall, AQP3 and AQP5 contribute to PDAC progression and metastasis, highlighting their potential as therapeutic targets and the need to identify potent and selective AQP3 and AQP5 inhibitors.

The reported involvement of AQP3 and AQP5 in PDAC two‐dimensional (2D) cell cultures and patient biopsies prompted us to investigate the impact of these proteins on the development of PDAC 3D cell cultures, which offer significant advantages over 2D cell cultures by establishing cell–cell and cell‐extracellular matrix interactions and closely mimicking the complexity of the tumor microenvironment [21, 22]. Spheroids provide a more physiologically relevant model for anticancer drug development due to their similarity to *in vivo* conditions [22, 23].

To investigate the implication of AQPs on PDAC spheroids' development, we chose two PDAC cell lines, BxPC‐3 and MiaPaca‐2, both derived from human PDAC primary tumors, with a moderate to poor differentiation [24]. These two cell lines were selected since BxPC‐3 exhibits high expression of AQP3 and AQP5 [18, 19], making it an ideal model to study their role in PDAC, while MiaPaca‐2 was described to recapitulate PDAC tumor progression and metastasis *in vivo* [25]. In addition, both have demonstrated their ability to form spheroids and mimic tumor microenvironment interactions [26, 27, 28], proving their suitability for 3D cell culture studies.

Thus, herein we generated spheroids from BxPC‐3 and MiaPaca‐2 cell lines and optimized growth conditions. Spheroids were treated with AQP3 inhibitors, and additionally, since no effective AQP5 inhibitors have been reported so far, we produced spheroids from AQP3‐ and AQP5‐knockdown cells to achieve a loss‐of‐function approach and identify the impact of these proteins on PDAC cancer biology.

## Materials and methods

### Compounds

The AQP3 inhibitor Auphen ([Au(phen)Cl_2_]Cl, phen = 1,10‐phenanthroline) was kindly provided by Prof. Angela Casini (Department of Chemistry, School of Natural Sciences, Technical University of Munich, Garching bei München, Germany). DFP00173 was purchased from Merck Life Science, Portugal. The stock solution concentrations were 10 mm for Auphen in water and 2.5 mm for DFP00173 in DMSO. Dilutions of the compounds were freshly prepared in aqueous solution before each experiment.

### Cell culture

BxPC‐3 cell line (ATCC: CRL‐1687) was cultured in Roswell Park Memorial Institute (RPMI) 1640 medium with high glucose (4.5 g/L), supplemented with 10% (v/v) fetal bovine serum (FBS, Gibco, Thermo Fisher Scientific, Waltham, MA, USA) at 37 °C under a 5% CO_2_ atmosphere. MiaPaca‐2 cell line (CRL‐1420) was grown in Dulbecco's modified Eagle's medium (DMEM) with high glucose (4.5 g/L) and supplemented with 10% FBS at 37 °C, 5% CO_2_ atmosphere. Cell lines were tested for mycoplasma contamination using the LookOut® mycoplasma Polymerase Chain Reaction Detection kit (Sigma‐Aldrich, Merck KGaA, Darmstadt, Germany).

### Transfection of BxPC‐3 cells

For AQP3 and AQP5 silencing, BxPC‐3 cells were transfected with a CRISPR‐Cas9 all‐in‐one plasmid expressing single‐guide RNAs (sgRNAs) targeting human AQP3 or human AQP5 (Genecopeia, Rockville, MD, USA), and copGFP to enable the selection of the transfected clones. For transfection, cells were seeded with a density of 80 000 cells cm^−2^ in six‐well plates. On the following day, CRISPR‐Cas9 plasmids were combined with EndoFectin™ Max (Genecopeia), following the manufacturer's protocol for BxPC‐3 cells transfection. Due to low transfection efficiency, AQP3 and AQP5 knockout cell lines could not be established even after clonal selection. Therefore, subsequent experiments were performed with AQP3 and AQP5 knockdown (KD) cells. Transfections were then validated at both mRNA and protein levels by quantitative PCR (qPCR) and western blot. All data regarding AQP3 or AQP5 KD BxPC‐3 cells were reported relative to nontransfected cells.

### RNA isolation, cDNA synthesis and qPCR

Total RNA was extracted from samples of BxPC‐3, BxPC‐3 AQP3 KD, BxPC‐3 AQP5 KD, and MiaPaca‐2 cells, using TRIzol reagent (Thermo Fisher Scientific, USA), according to the manufacturers' protocol [29]. RNA was quantified using Nanodrop 2000c spectrophotometer (Thermo Fisher Scientific, USA) and 1 μg of good quality RNA (260/280 nm and 260/230 nm ratios of 2) was used to synthesize cDNA with first‐strand cDNA synthesis kit (NZYtech, Lisbon, Portugal), according to the manufacturer's instructions. Transcript levels of human AQP1, AQP3, AQP5, AQP7, AQP8, AQP10, AQP11 and housekeeping gene hypoxanthine phosphoribosyltransferase 1 (HPRT‐1) were quantified through qPCR performed in a CFX96 Real‐Time System C1000 (BioRad, Hercules, CA, USA) using TaqMan Universal Master Mix II with UNG (Applied Biosystems, Thermo Fisher Scientific, USA) and the following specific and predesigned TaqMan Gene Expression Assays: AQP1 (Hs01028916_m1), AQP3 (Hs01105469_g1), AQP5 (Hs00387048_m1), AQP7 (Hs00357359_m1), AQP8 (Hs01086280_g1), AQP10 (Hs00369738_m1), AQP11 (Hs005426181_m1) and HPRT‐1 (Hs02800695_m1). The cDNA was amplified in the following conditions: 50 °C for 2 min, 95 °C for 10 min, followed by 40 cycles of 15 s at 95 °C and 1 min at 60 °C. AQP gene expression was normalized to the housekeeping values and relative quantification was determined using a variation of the Livak method [30]. Samples were run in triplicate.

### Protein extraction, quantification, and western blot

Protein extraction from BxPC‐3, BxPC‐3 AQP3 KD, BxPC‐3 AQP5 KD, and MiaPaca‐2 cells was performed using Mem‐PER™ Plus Membrane Protein Extraction Kit (Thermo Fisher Scientific, USA), according to the manufacturer's instructions. The membrane fraction of the protein lysates was quantified using Pierce™ BCA Protein Assay kit (Thermo Fisher Scientific, USA), and 20 μg of total protein of each lysate was mixed with Laemmli buffer and heated at 95 °C for 5 min before performing SDS/PAGE. After electrophoresis, proteins were then transferred to polyvinylidene difluoride (PVDF) membranes (BioRad, Hercules, CA, USA). Membranes were blocked with 3% (w/v) bovine serum albumin (BSA) in tris buffered saline with tween (TBS‐T) solution before overnight incubation at 4 °C with primary antibodies: mouse anti‐AQP3 (dilution 1:100, Santa Cruz Biotechnologies), mouse anti‐AQP5 (dilution 1:200, Santa Cruz Biotechnologies), and mouse anti‐α‐tubulin (dilution 1:1000, Sigma) diluted in 3% (w/v) BSA in TBS‐T. The next day, membranes were incubated at room temperature with donkey anti‐mouse secondary antibody (dilution 1:3000; Jackson ImmunoResearch Laboratories, West Grove, PA, USA) with horseradish peroxidase for enhanced chemiluminescence (ECL) detection with SuperSignal™ West Femto Maximum Sensitivity Substrate (Thermo Fisher Scientific, USA) using iBright™ CL750 Imaging System (Thermo Fisher Scientific, USA) [31]. The resulting bands were quantified using the imagej software (https://imagej.net/ij/) and normalized to the α‐tubulin values.

### Water and glycerol permeability assays

For water and glycerol permeability assays, BxPC‐3 and MiaPaca‐2 cells were centrifuged at 150 × g for 5 min and the pellet was resuspended in phosphate buffered saline (PBS). The diameter of cells was measured under the light microscope using the ImageJ software (https://imagej.net/ij/), considering a spherical shape and a homogeneous cell suspension. Permeability assays were performed by stopped‐flow light scattering on a HI‐TECH Scientific PQ/SF‐53 stopped‐flow apparatus, with a 2‐ms dead time, temperature controlled and interfaced with a microcomputer. For each run, cell suspensions (0.1 mL) were challenged with an equal volume of hyperosmotic solutions at 23 °C and the time course of volume changes was measured by measuring the 90° scattered light intensity at 400 nm. For each experimental condition, three to eight runs were analyzed. For the measurement of water permeability (P_f_), cells were challenged with a hyperosmotic solution containing mannitol (450 mm in PBS, pH 7.4), which is a nonpermeant solute, leading to water efflux and subsequent cell shrinkage. For glycerol permeability (P_gly_) assays, cells were challenged with a hyperosmotic solution containing glycerol (450 mm in PBS, pH 7.4), creating an inwardly directed glycerol gradient. After a fast cell shrinkage due to water efflux, glycerol enters the cell in response to its chemical gradient followed by water influx and consequent cell reswelling. Baselines were acquired using PBS as the isotonic shock solution. P_f_ was determined by:

(1) $$P_{f}=k\frac{V_{0}}{A}\frac{1}{V_{w}{\left(\mathrm{os}m_{\text{out}}\right)}_{\infty }}$$

where *V*_w_ is the molar volume of water, *V*_0_/*A* is the initial cell volume to area ratio, (osm_out_)_∞_ is the final medium osmolarity after the applied osmotic gradient, and *k* (s^−1^) is the single exponential time constant fitted to the light scattering of cells' shrinkage. *P*_gly_ was calculated by:

(2) $$P_{\mathrm{gly}}=k\frac{V_{0}}{A}$$

where *k* is the single exponential time constant fitted to the light scattering signal of glycerol influx [32].

### Cytotoxicity assays

The cytotoxicity of AQP3 inhibitors Auphen and DFP00173 was evaluated using CellTiter‐Glo 3D® assay (Promega, Madison, WI, USA) according to the manufacturer's instructions. MiaPaca‐2 and BxPC‐3 cells were inoculated at a density of 20 000 cells per well in 96‐well plates and grown at 37 °C, 5% CO_2_ until reaching 80% confluence. Cells were then incubated for 24 h with the compounds at different concentrations: 0, 2.5, 5, 10, 12.5, 15, 20, 25 μm for Auphen and 0, 2.5, 5, 10, 12.5, 15, 20, 25, 40, 50 μm for DFP00173. After treatment, cells were incubated for 15 min with CellTiter‐Glo 3D® with agitation and the luminescence was measured using a microplate reader (Varioskan™ LUX multimode microplate reader, Thermo Scientific). Samples were run in triplicate.

### Spheroids' establishment and monitoring of growth

For spheroids' formation with Matrigel®, cells were seeded in Nunclon™ Sphera™ ultra‐low attachment 96‐well plates (Thermo Fisher Scientific, USA) at a density of 3500 cells per well for BxPC‐3, BxPC‐3 AQP3 KD and BxPC‐3 AQP5 KD cells, and 1250 cells per well for MiaPaca‐2 cells. Cells were resuspended with Growth Factor Reduced Matrigel® (final concentration of 0.4 mg mL^−1^, Corning, Merck) and plates were centrifuged at 370 × g for 5 min at 4 °C. Plates were then incubated at 37 °C, 5% CO_2_. Spheroids' growth was monitored for 7 days using a Primovert Inverted ZEISS Microscope with a × 4 objective, with an integrated HDcam camera and using the ZEN 3.5 (blue edition) software [33]. For each experimental condition, three spheroids were imaged from Day 1 to Day 7. The spheroids' mean diameter, area and perimeter were measured using the software SpheroidSizer [34]. The circularity was calculated according to the equation below [33]:

(3) $$\text{Circularity}=\frac{4\pi \times \text{Area}}{\text{Perimete}r^{2}}$$

### Spheroids' treatment and viability assay

Three‐day‐old BxPC‐3 and MiaPaca‐2 spheroids were treated for 24 h with 5 μm Auphen or 25 μm DFP00173, prepared in culture medium. The following day, the media were replaced, and spheroids were allowed to grow until day 7.

During the optimization of BxPC‐3 and MiaPaca‐2 spheroids' formation, cell viability was assessed on Day 3 and Day 7 using the CellTiter‐Glo® 3D cell viability assay. Three spheroids per condition were incubated with CellTiter‐Glo® 3D with agitation for 15 min and luminescence was measured using a microplate reader (Varioskan™ LUX multimode microplate reader, Thermo Scientific). For spheroids treated with AQP3 inhibitors or silenced for AQP3 or AQP5, spheroids' viability was evaluated at Day 4 and Day 7 using the same technique to allow accurate comparisons.

### Statistical analysis

The results were expressed as individual values and mean ± standard deviation (SD) of three independent experiments. Data were analyzed either by unpaired Student's *t*‐test or two‐way ANOVA when comparing two independent variables using the GraphPad Prism software 8. A *P* < 0.05 was considered statistically significant.

## Results

### Assessment of AQP expression and function on pancreatic ductal adenocarcinoma cell lines

**Fig. 1:**
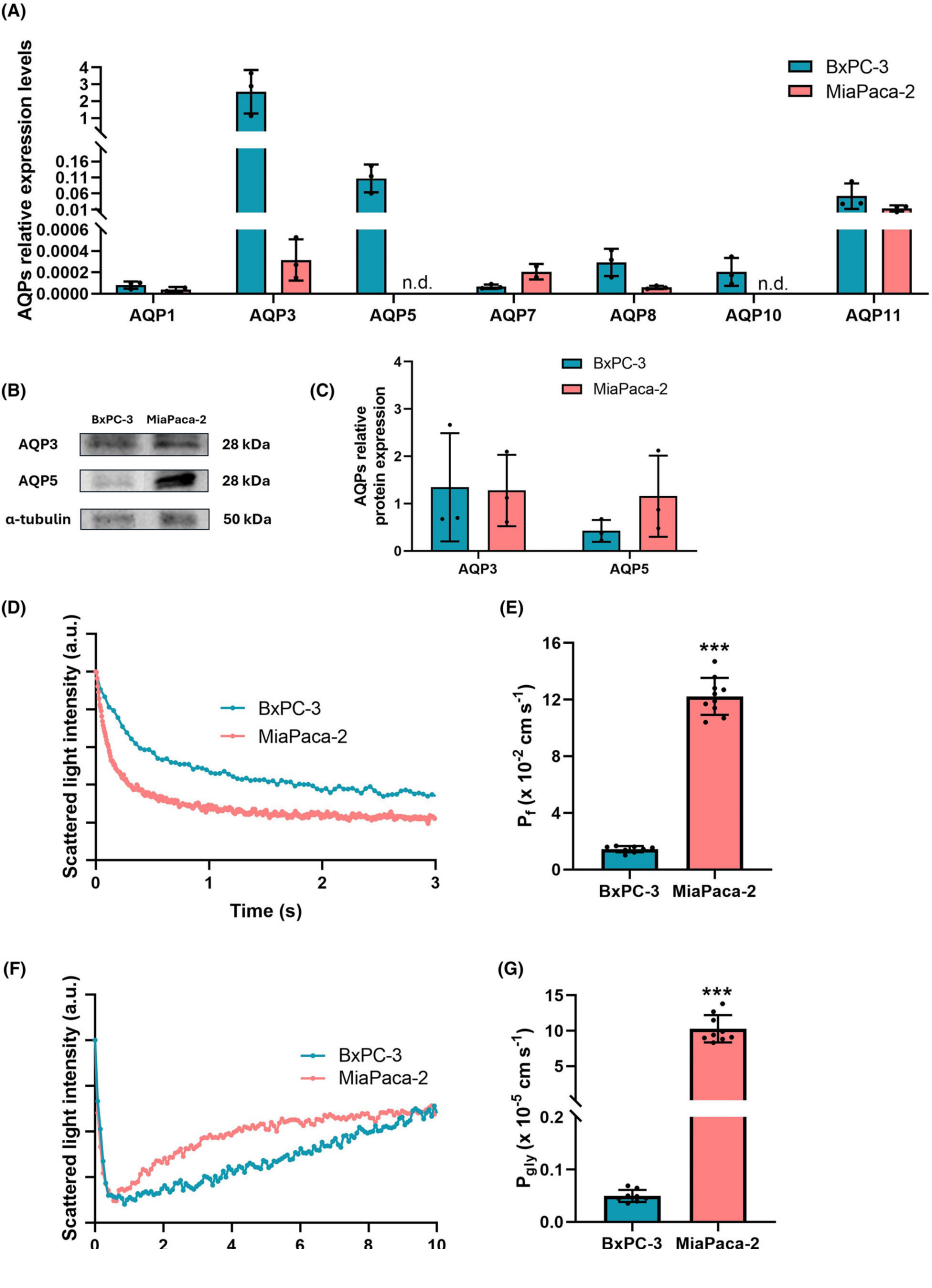
Evaluation of AQPs expression and function on BxPC‐3 and MiaPaca‐2 cells. (A) Relative AQPs mRNA expression in BxPC‐3 and MiaPaca‐2 cells, normalized to HPRT‐1 mRNA expression. (B) Representative blots exhibiting AQP3, AQP5, and α‐tubulin bands for BxPC‐3 and MiaPaca‐2 cells. (C) Protein expression levels of AQP3 and AQP5 in BxPC‐3 and MiaPaca‐2 cells, normalized to α‐tubulin expression. (D) Representative stopped‐flow light scattering signal of BxPC‐3 and MiaPaca‐2 cells challenged with a hyperosmotic mannitol solution to assess water permeability. Cells exposed to a hyperosmotic mannitol solution shrink due to water efflux. (E) Water permeability (P_f_) coefficients for BxPC‐3 and MiaPaca‐2 cells. (F) Representative stopped‐flow light scattering signal of BxPC‐3 and MiaPaca‐2 cells challenged with a hyperosmotic glycerol solution to evaluate glycerol permeability via aquaglyceroporins. Cells were exposed to a hyperosmotic glycerol solution leading to a first water efflux followed by glycerol and water influx, with consequent cell reswelling. (G) Glycerol permeability (P_gly_) coefficients for BxPC‐3 and MiaPaca‐2 cells. Data are presented as mean ± SD of three independent experiments together with individual data points obtained from three biological replicates, all used for statistical analysis by unpaired Student's *t*‐test. Asterisks indicate significant differences, *P < 0.05, control *vs*. ovxAQP cells. n.d.—not detected. ***P < 0.001, BxPC‐3 *vs*. MiaPaca‐2 cells.

Prior to establishing PDAC 3D cell cultures, a screening of AQP mRNA levels in BxPC‐3 and MiaPaca‐2 cells was performed using quantitative PCR (qPCR). Results showed that AQP3 and AQP5 were the most abundant AQP isoforms in BxPC‐3 cells, while for MiaPaca‐2 cells, AQP3 expression was lower and AQP5 was undetectable. Both PDAC cell lines showed similar transcript levels of AQP11, whereas AQP1, AQP7, AQP8 and AQP10 were detected in lower amounts, with AQP10 not being expressed in MiaPaca‐2 cells (Fig. 1A). After assessing AQP expression in these cell lines, we evaluated the protein levels of AQP3 and AQP5, the isoforms most frequently overexpressed in PDAC [6], through western blot. AQP3 was expressed at similar levels in both cell lines, whereas AQP5 protein levels were higher in MiaPaca‐2 cells than in BxPC‐3 cells, although the difference was not statistically significant. Given that AQP5 mRNA expression was undetected in MiaPaca‐2 cells, this could indicate a potential higher mRNA degradation (Fig. 1B,C and Fig. S1).

In order to characterize AQP activity, permeability assays of BxPC‐3 and MiaPaca‐2 cells were performed using stopped‐flow light scattering. To assess water permeability (P_f_), cells were challenged with a hyperosmotic mannitol solution, a nonpermeant solute, resulting in water outflow and cell shrinkage (Fig. 1D). The rate of cell shrinkage was faster for MiaPaca‐2 cells, which exhibited an 8.5‐fold larger water permeability than for BxPC‐3 cells, which aligns well with the higher AQP5 protein expression detected (Fig. 1E).

For the evaluation of glycerol permeability, cells were exposed to a hyperosmotic glycerol solution leading to a fast water efflux, followed by glycerol and water influx via aquaglyceroporins, resulting in cell reswelling (Fig. 1F). A higher glycerol permeation was observed for MiaPaca‐2 cells compared to BxPC‐3 cells, with a 206‐fold difference. The higher glycerol fluxes observed for MiaPaca‐2 cells may be attributed to the aquaglyceroporin activity of both AQP3 and AQP7, since AQP7 was also detected at the mRNA level in these cells. (Fig. 1G).

### Optimization of BxPC‐3 and MiaPaca‐2 spheroids establishment

**Fig. 2:**
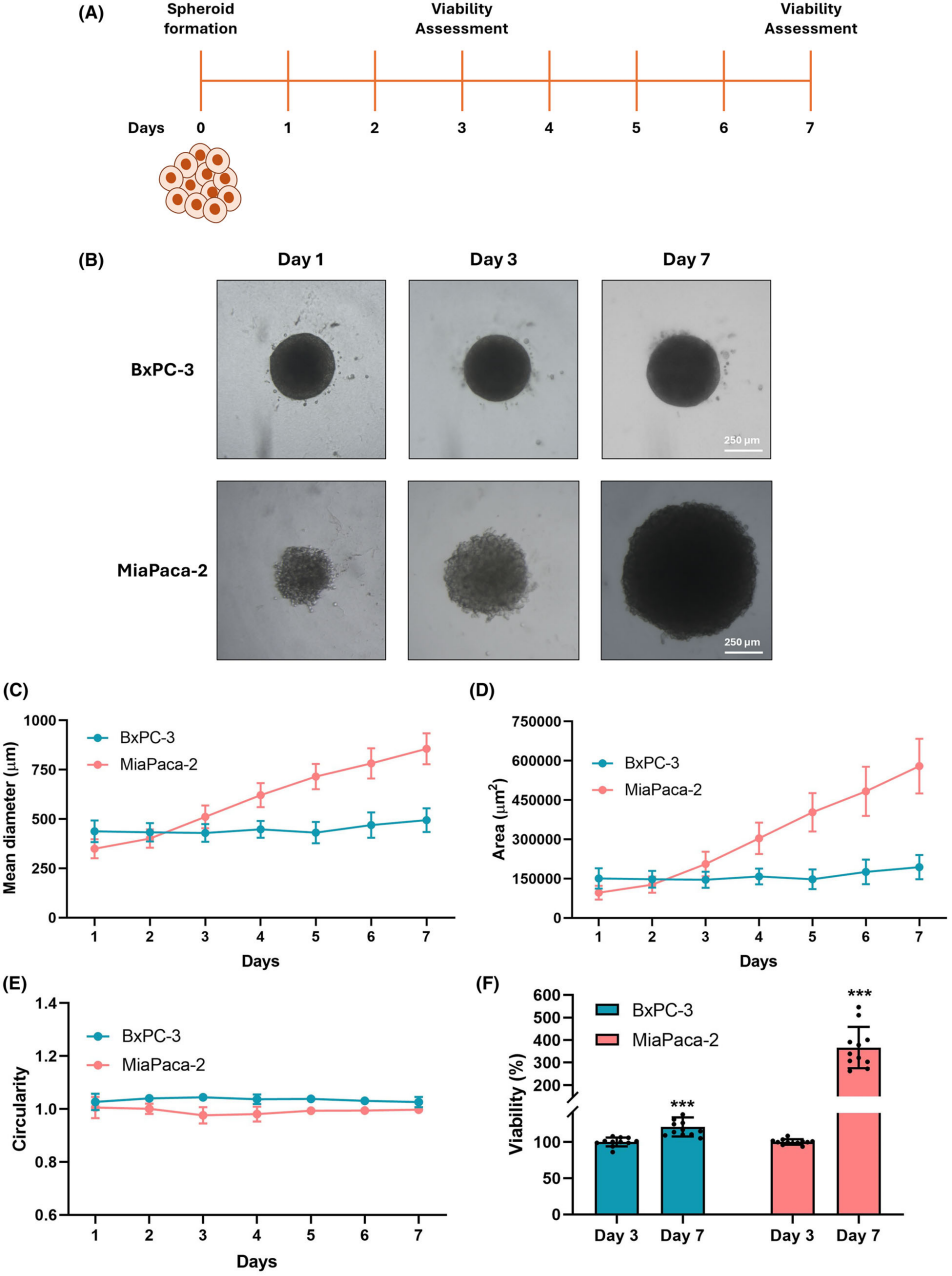
BxPC‐3 and MiaPaca‐2 spheroids' formation with Matrigel. (A) Schematic representation of the experimental design for BxPC‐3 and MiaPaca‐2 spheroids' formation and viability assessment for 7 days. (B) Representative microscope images of BxPC‐3 and MiaPaca‐2 spheroids on Days 1, 3, and 7, formed with Matrigel. Scale bar: 250 μm. BxPC‐3 and MiaPaca‐2 mean spheroids' diameter (C), area (D), and circularity (E) as a function of the number of days in culture. (F) Cell viability evaluated with CellTiter‐Glo® assay for BxPC‐3 and MiaPaca‐2 spheroids on Day 3 and Day 7, normalized to the cell viability on Day 3. Data are presented as mean ± SD of three independent experiments together with individual data points obtained from three biological replicates, all used for statistical analysis by unpaired Student's *t*‐test. Asterisks indicate significant differences, ***P < 0.001, Day 3 *vs*. Day 7.

After confirming AQP3 and AQP5 expression and function in BxPC‐3 and MiaPaca‐2 cells, we proceeded with the optimization of spheroids' establishment for these two cell lines using Matrigel®, commonly used as an extracellular matrix mimetic [35]. Cells were seeded with Matrigel® in ultra‐low attachment 96‐well plates and centrifuged to promote spheroids' formation (Day 0). Spheroids were imaged from Day 1 to Day 7, and viability was evaluated on Day 3 and Day 7 to ensure the maintenance of viable spheroids throughout the experiment (Fig. 2A).

Our criteria for optimal spheroids' formation for subsequent studies required that, by Day 3, spheroids were fully formed and compact, with diameters ranging between 300 and 500 μm, indicative of an optimal balance of nutrients and oxygen without hypoxia or necrotic core, ensuring suitability for drug testing [36, 37]. In addition, spheroids needed to remain viable until Day 7 to provide a sufficient time window to observe differences in spheroids' growth and circularity.

BxPC‐3 cells formed well delimitated and compact spheroids, with appropriate mean diameter on Day 3 (Fig. 2B–E). Spheroids' viability increased 21% on Day 7 compared to Day 3 (Fig. 2F), reflecting BxPC‐3 spheroids' proliferation, aligned with the increase in both diameter and area over the course of the experiment. MiaPaca‐2 cells formed spheroids not as compact as BxPC‐3, although still structured with optimal circularity, and with adequate diameter on Day 3. MiaPaca‐2 spheroids remained viable throughout the experiment, exhibiting a 267% increase in viability on Day 7 compared to Day 3. This enhanced spheroids' proliferation is consistent with the augmented mean diameter and area, indicating a higher spheroids' growth than for BxPC‐3 cells. Therefore, spheroids' establishment with Matrigel proved to be a suitable method to generate PDAC models from BxPC‐3 and MiaPaca‐2 cells.

### The AQP3 inhibitors Auphen and DFP00173 affect PDAC spheroids' development

**Fig. 3:**
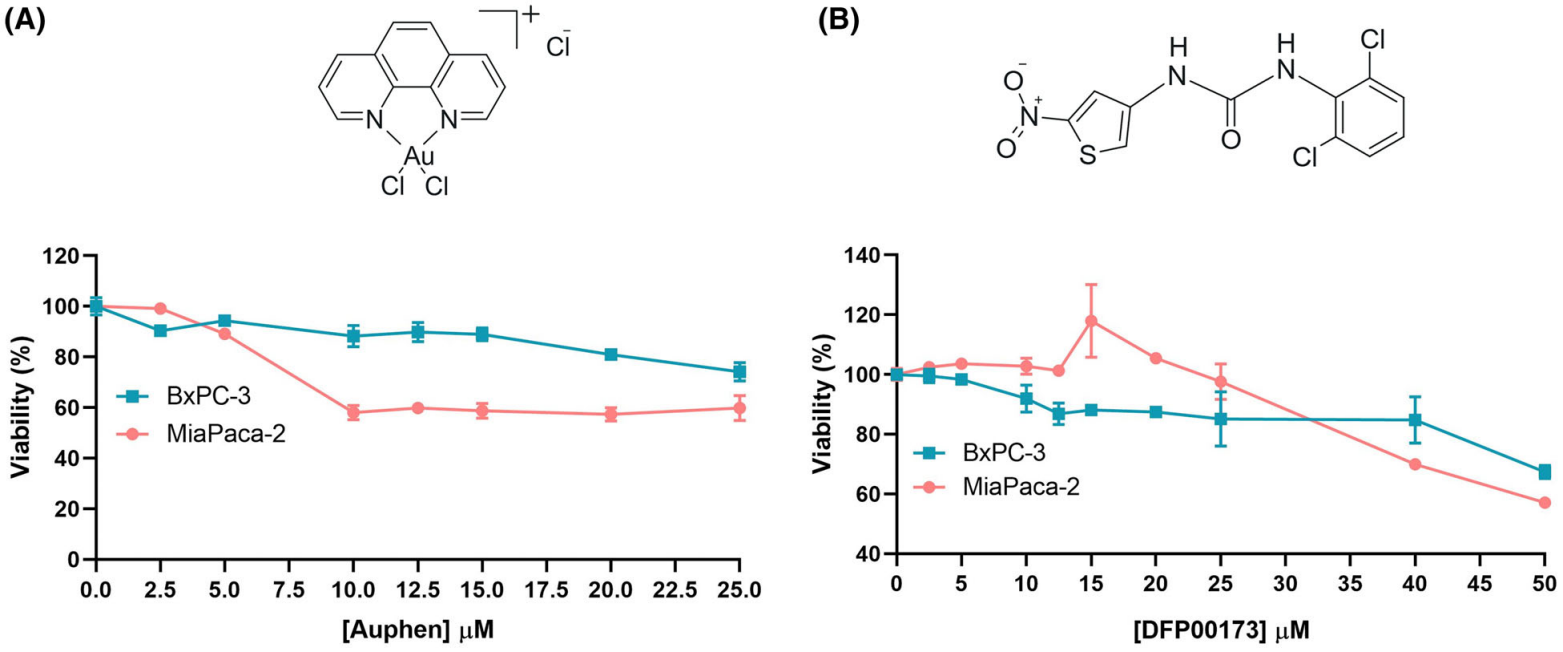
Evaluation of Auphen and DFP00173 cytotoxicity on BxPC‐3 and MiaPaca‐2 cells. Cell viability assessed using CellTiter‐Glo® assay after treating BxPC‐3 and MiaPaca‐2 cells for 24 h with increasing concentrations of Auphen (A) and (B) DFP00173 (0–50 μm), and respective chemical structures. Data are presented as mean ± SD of three independent experiments.

To elucidate the role of AQP3 and AQP5 in the development of PDAC spheroids, our first approach was targeting these AQPs with specific inhibitors. Given the lack of effective AQP5 inhibitors, we focused on AQP3 inhibitors—Auphen and DFP00173—to assess the contribution of AQP3 in PDAC spheroids' growth. Auphen and DFP00173 have been reported as potent AQP3 inhibitors, with IC_50_ values of 0.8 μm [32] and 0.2 μm, respectively, in red blood cells [38]. Before assessing the effect of AQP3 inhibitors on PDAC spheroids, we evaluated their cytotoxicity in 2D cell cultures to determine the maximum nontoxic concentration to be used in the subsequent experiments in 3D cell cultures. BxPC‐3 and MiaPaca‐2 cells were treated with different concentrations (0–50 μm) of Auphen and DFP00173 for 24 h, after which cell viability was assessed. Interestingly, the organogold compound exhibited greater toxicity toward MiaPaca‐2 cells than BxPC‐3 cells. MiaPaca‐2 cell viability was reduced to 58% when treated with Auphen at concentrations above 5 μM. No significant cytotoxic effects were observed for BxPC‐3 cells, except when treated with 25 μm Auphen (Fig. 3A,B). Regarding DFP00173 cytotoxicity, MiaPaca‐2 cells showed a 30% reduction in viability when exposed to concentrations above 25 μm, while BxPC‐3 cells experienced a 33% reduction in viability only at 50 μm (Fig. 3C). Thus, the selected concentrations for subsequent experiments ensuring cell viability above 75% in both BxPC‐3 and MiaPaca‐2 cells were 5 μm for Auphen and 25 μm for DFP00173.

**Fig. 4:**
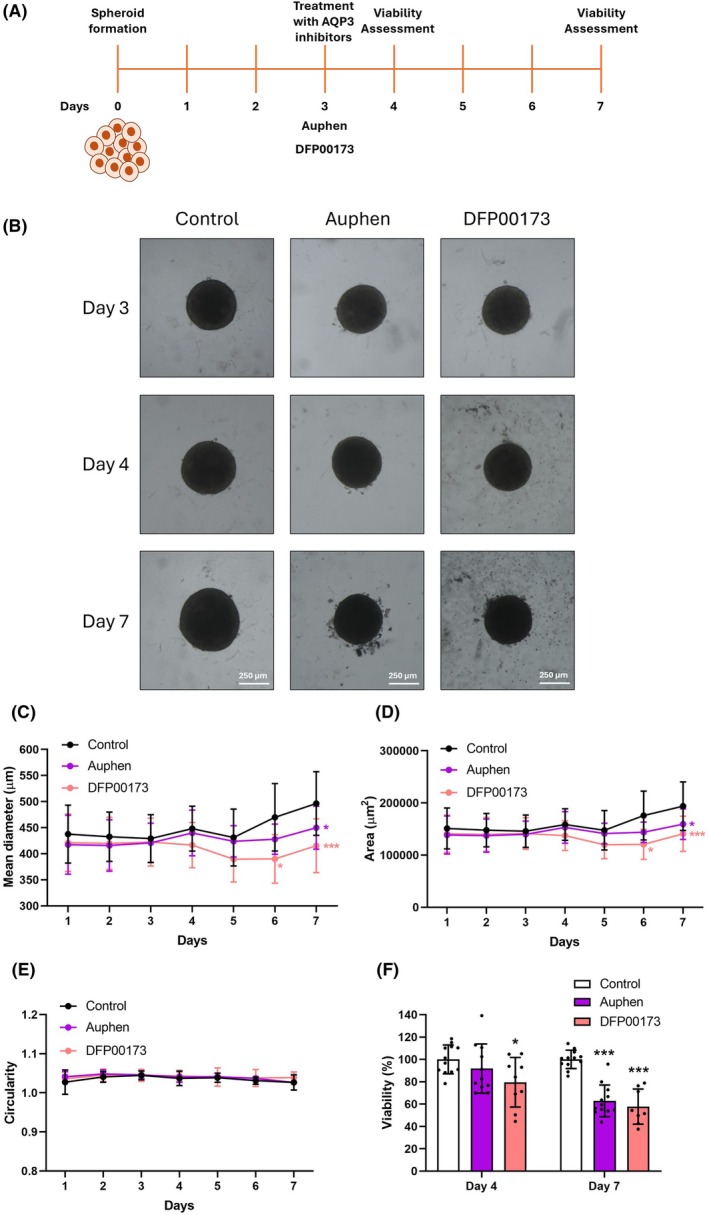
Effect of Auphen and DFP00173 in BxPC‐3 spheroids. (A) Schematic representation of the experimental design for BxPC‐3 spheroids' formation, treatment with AQP3 inhibitors (Auphen and DFP00173) and viability assessment, for a total of 7 days. (B) Representative microscope images of BxPC‐3 spheroids on Days 3, 4, and 7 nontreated and treated with Auphen or DFP00173. Scale bar: 250 μm. BxPC‐3 mean spheroids' diameter (C), area (D), and circularity (E) as a function of the number of days in culture, for spheroids non‐treated and treated with Auphen or DFP00173. (F) BxPC‐3 spheroids' viability was measured using CellTiter‐Glo® 3D assay on Days 4 and 7 for spheroids non‐treated and treated with Auphen or DFP00173, normalized to the viability of control spheroids. Data are presented as mean ± SD of three independent experiments together with individual data points obtained from three biological replicates, all used for statistical analysis. Significance was analyzed by ordinary one‐way ANOVA and Tukey's test. Asterisks indicate significant differences, *P < 0.05, ***P < 0.001, non‐treated *vs*. treated spheroids.

BxPC‐3 spheroids were treated on day 3 with AQP3 inhibitors for 24 h, and spheroids' viability was evaluated on Days 4 and 7 (Fig. 4A). Treatment with Auphen reduced spheroids' diameter and area by Day 7, without affecting circularity, although cell dissociation from the spheroid was detected (Fig. 4B–E). DFP00173 treatment resulted in a more pronounced decrease in spheroids' diameter and area compared to Auphen, observed on both Days 6 and 7, with spheroids also showing noticeable cell dissociation. Regarding the viability assessment, DFP00173 diminished spheroids' viability by 20% on Day 4 while on Day 7, both Auphen and DFP00173 reduced viability by 37% and 42%, respectively. These findings, along with the observed reductions in spheroids' diameter and area, suggest that the impaired spheroids' viability could be attributable to disrupted spheroids' growth, probably via AQP3 inhibition.

**Fig. 5:**
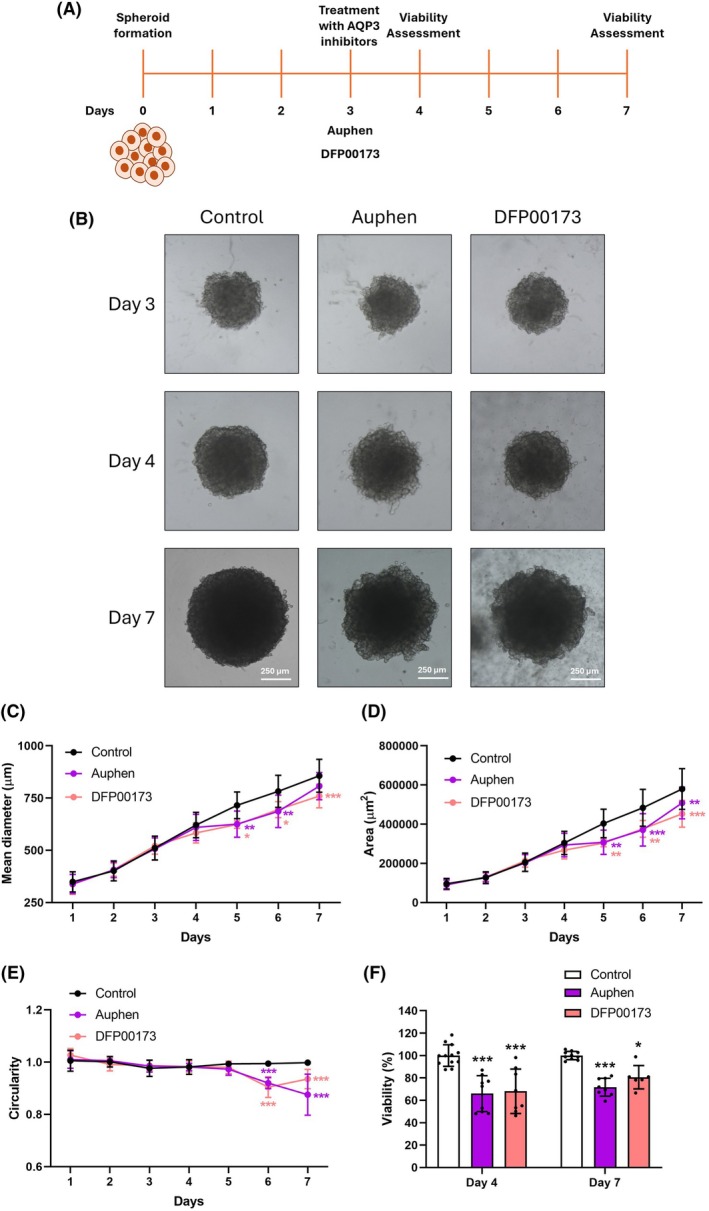
Effect of Auphen and DFP00173 in MiaPaca‐2 spheroids. (A) Schematic representation of the experimental design for MiaPaca‐2 spheroids' formation, treatment with AQP3 inhibitors (Auphen and DFP00173) and viability assessment, for a total of 7 days. (B) Representative microscope images of MiaPaca‐2 spheroids on Days 3, 4, and 7 nontreated and treated with Auphen or DFP00173. Scale bar: 250 μm. MiaPaca‐2 mean spheroids' diameter (C), area (D), and circularity (E) as a function of the number of days in culture, for spheroids nontreated and treated with Auphen or DFP00173. (F) MiaPaca‐2 spheroids' viability was measured using CellTiter‐Glo® 3D assay on Day 4 and Day 7 for spheroids nontreated and treated with Auphen or DFP00173, normalized to the viability of control spheroids. Data are presented as mean ± SD of three independent experiments together with individual data points obtained from three biological replicates, all used for statistical analysis. Significance was analyzed by ordinary one‐way ANOVA and Tukey's test. Asterisks indicate significant differences, *P < 0.05, **P < 0.01. ***P < 0.001, non‐treated *vs*. treated spheroids.

MiaPaca‐2 spheroids were also treated with AQP3 inhibitors on Day 3 for 24 h, with viability assessed on Days 4 and 7 (Fig. 5A). Treatment with Auphen led to the reduction in spheroids' diameter and area on Days 5 and 6, with spheroids' area further diminished on Day 7, compared to control spheroids (Fig. 5B–D). Spheroid circularity was also reduced on Days 6 and 7 (Fig. 5E). Similar effects were observed with DFP00173, which decreased spheroids' diameter and area from Days 5 to 7, along with decreased circularity on Days 6 and 7. Representative images from Day 7 highlight these differences upon treatment with Auphen or DFP00173, with spheroids appearing smaller, less compact with poorly defined borders and distinguishable cells at the periphery.

When analyzing the effect of Auphen and DFP00173 on viability, a decrease on Day 4 of 34% and 32% was detected, while on Day 7, Auphen and DFP00173 treatment reduced spheroids' viability by 28% and 19%, respectively (Fig. 5F). Although no differences were evident on day 4 for MiaPaca‐2 spheroids' diameter, area, and circularity, spheroids treated with Auphen and DFP00173 exhibited irregular edges with distinguishable cells at the borders, lower cell density, and a smaller dark core than control spheroids, suggesting early signs of diminished spheroids' growth that became more pronounced on Day 7. Therefore, the decreased spheroids' viability induced by these AQP3 inhibitors could reflect impaired spheroids' growth through AQP3 blockage, aligning with the observed reductions in spheroids' diameter, area, and circularity on day 7.

### AQP3 and AQP5 knockdown impairs BxPC‐3 spheroids' development

To further investigate the contribution of AQP3 and AQP5 in the development of PDAC spheroids' development, we employed a loss‐of‐function strategy using the CRISPR‐Cas9 technique.

MiaPaca‐2 cells presented very low transfection efficiency with CRISPR‐Cas9, likely attributable to the cell lines' phenotypic diversity and genomic heterogeneity [39]. As a result, we proceeded with the AQP3 and AQP5 silencing in BxPC‐3 cells and since complete knockout was not achieved, we generated BxPC‐3 derivatives with AQP3 or AQP5 knockdown (AQP3 KD and AQP5 KD).

**Fig. 6:**
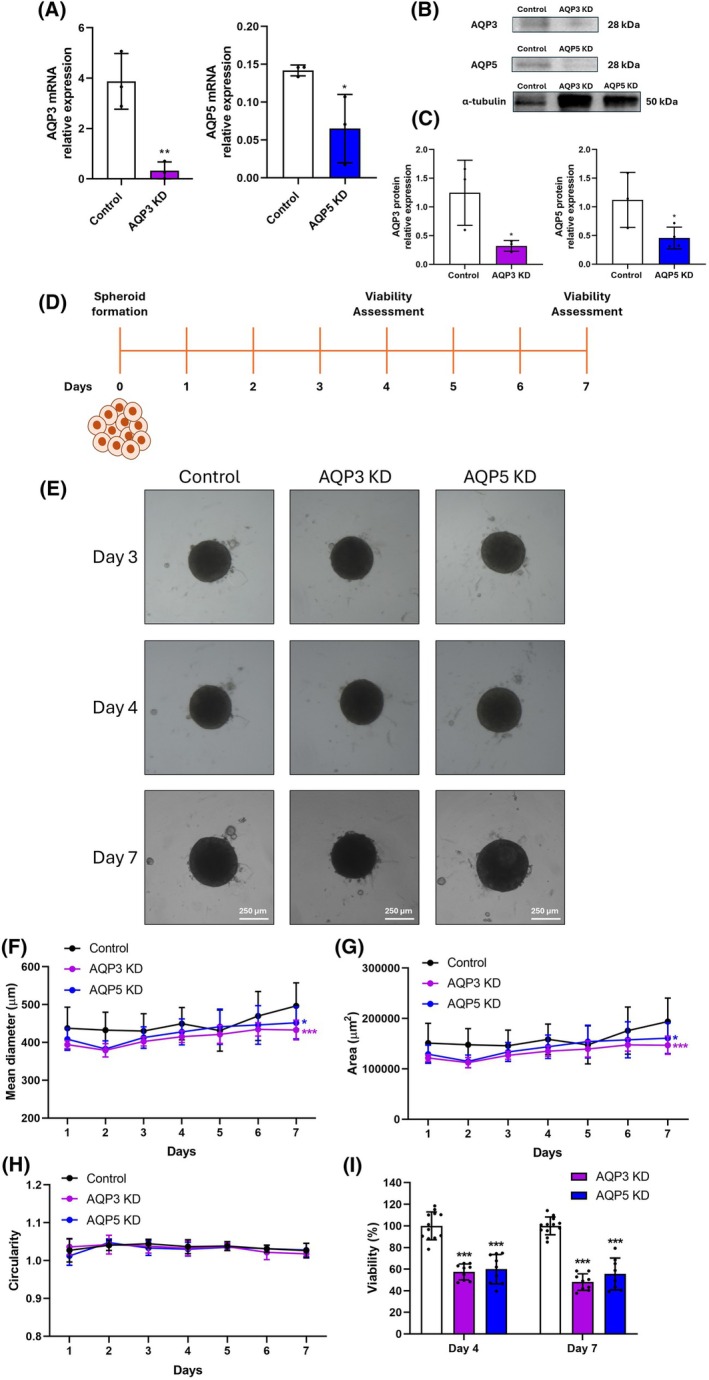
Impact of AQP3 and AQP5 silencing in BxPC‐3 spheroids. (A) Relative mRNA expression of AQP3 and AQP5 in BxPC‐3 cells control and knockdown for AQP3 (AQP3 KD) or AQP5 (AQP5 KD), normalized to HPRT‐1 mRNA expression. (B) Representative blots exhibiting AQP3, AQP5, and α‐tubulin bands for control, AQP3 KD, and AQP5 KD cells. (C) Protein expression levels of AQP3 and AQP5 in control, AQP3 KD, and AQP5 KD cells, normalized to α‐tubulin expression. (D) Schematic representation of the experimental design for control, AQP3 KD, and AQP5 KD spheroids' formation and viability assessment, for a total of 7 days. (E) Representative microscope images of control, AQP3 KD, and AQP5 KD spheroids on day 3, 4, and 7. Scale bar: 250 μm. Control, AQP3 KD, and AQP5 KD mean spheroids' diameter (F), area (G), and circularity (H) as a function of the number of days in culture. (I) Control, AQP3 KD, and AQP5 KD spheroid viability was measured using CellTiter‐Glo® 3D assay on Day 4 and Day 7, normalized to the viability of control spheroids. Data are presented as mean ± SD of 3 independent experiments together with individual data points obtained from three biological replicates, all used for statistical analysis. Significance was analyzed by ordinary one‐way ANOVA and Tukey's test. Asterisks indicate significant differences, *P < 0.05, **P < 0.01, ***P < 0.001, control *vs*. AQP3 or AQP5 KD spheroids. Schematic representation of the role of aquaporin‐3 (AQP3) and aquaporin‐5 (AQP5) in pancreatic ductal adenocarcinoma (PDAC). Both proteins are upregulated in PDAC and are associated with tumor progression and metastatic potential. Silencing AQP3 or AQP5 in PDAC spheroids results in decreased diameter, area, and overall growth, underscoring their key contribution to PDAC development.

Prior to the 3D cell culture assays, we validated AQP3 and AQP5 mRNA and protein expression in the parental BxPC‐3, AQP3 KD, and AQP5 KD cell lines by performing qPCR and western blot, respectively. Our data revealed an 11.7‐fold reduction in AQP3 mRNA expression in AQP3 KD cells and a 2.2‐fold decrease in AQP5 mRNA levels in AQP5 KD cells, compared to control cells (Fig. 6A). In addition, AQP3 and AQP5 protein expression was decreased in AQP3 KD and AQP5 KD cells (5.2‐fold and 2.8‐fold decrease, respectively), confirming the silencing of AQP3 and AQP5 in BxPC‐3 cells (Fig. 6B,C and Fig. S2).

Following the validation of AQP3 and AQP5 expression on BxPC‐3 AQP3 KD and AQP5 KD cells, the establishment of 3D spheroids from these derivatives was initiated to evaluate the impact of AQP3 and AQP5 on PDAC spheroids' development. Spheroids' formation in BxPC‐3 control and silenced cells was monitored over 7 days, with viability assessed on Days 4 and 7 to enable comparisons with spheroid assays involving AQP3 inhibitors (Fig. 6D). AQP3 KD and AQP5 KD spheroids exhibited decreased diameter and area by Day 7, being more pronounced in AQP3 KD spheroids, while circularity remained unchanged (Fig. 6E–H). Spheroids' viability was significantly impaired in AQP3 KD and AQP5 KD spheroids on Day 4 by 43% and 52% and on Day 7 by 40% and 45%, respectively (Fig. 6I).

By Day 4, the unaltered spheroids' diameter and area, along with the preserved spheroid structure, could indicate that the reduced spheroids' viability may be caused by cell death within the spheroid, potentially leading to the formation of a necrotic core. However, by Day 7, a reduction in spheroids' diameter and area was observed as well as cell dissociation from the spheroid, suggesting that the loss of viability could be attributed to impaired spheroids' growth and increased cell death (Fig. 6E and Fig. S3). Comparing AQP3 KD spheroids with control spheroids treated with Auphen or DFP00173, both exhibited decreased spheroids' diameter and area on Day 7, with no differences in circularity. Moreover, both models showed impaired spheroids' viability and cell dissociation from the spheroid, emphasizing the selectivity of these compounds toward AQP3 and the critical role of this AQP in PDAC spheroids' development. Additionally, the behavior of AQP5 KD spheroids closely resembled AQP3 KD spheroids, reinforcing the relevance of both AQP3 and AQP5 in PDAC progression.

## Discussion

The overexpression of AQP3 and AQP5 has been associated with PDAC development and metastasis, contributing to key processes for tumorigenesis [6]. Studies with PDAC cells silenced for AQP3 and/or AQP5 revealed the important role of these AQPs in promoting cell migration [18, 19], influencing cell morphology and cell–cell adhesion, with only AQP5 impacting cell stiffness and membrane fluidity [19]. Furthermore, AQP3 was found to promote cell growth and inhibit apoptosis in PDAC [15], whereas AQP5 facilitated cell invasion [16]. In PDAC biopsies, both AQP3 and AQP5 were overexpressed in early stages, contributing to EMT and tumorigenesis [17]. However, in advanced stages, AQP3 expression increased whereas AQP5 was undetected [17], which may be considered a prognosis biomarker.

Considering the reported crucial role of AQP3 and AQP5 in PDAC progression, we studied their contribution to PDAC spheroids' development through the use of AQP3 inhibitors and silencing AQP3 or AQP5 gene expression. For that purpose, we evaluated both their mRNA and protein levels in the PDAC cell lines BxPC‐3 and MiaPaca‐2. AQP3 mRNA expression was higher for BxPC‐3 cells, but similar levels were detected at the protein level for the two cell lines. AQP5 protein expression was elevated in MiaPaca‐2 compared to BxPC‐3 cells, although the difference was not statistically significant. In contrast, AQP5 mRNA expression was more abundant in BxPC‐3 cells and not detected in MiaPaca‐2 cells, suggesting a possible increase in mRNA degradation in MiaPaca‐2 cells. Both water and glycerol permeability were higher in MiaPaca‐2 cells, likely attributed to AQP5 protein expression and AQP3‐ and AQP7‐mediated glycerol fluxes.

After the validation of AQP3 and AQP5 expression and function, we optimized PDAC spheroids' establishment for BxPC‐3 and MiaPaca‐2 cells using Matrigel® in ultra‐low attachment 96‐well plates, followed by centrifugation. BxPC‐3 cells were able to form compact spheroids with well‐defined borders and a mean diameter between 300 and 500 μm on day 3, exhibiting a 21% increase in spheroids' proliferation between Days 3 and 7. MiaPaca‐2 cells generated spheroids with adequate diameter by Day 3, demonstrating optimal circularity, although these spheroids were less compact compared to BxPC‐3 spheroids. MiaPaca‐2 spheroids presented a significantly higher spheroids' proliferation than BxPC‐3, with a 267% increase in spheroids' growth from Day 3 to 7. This method proved to be effective for creating viable and compact PDAC spheroids from both BxPC‐3 and MiaPaca‐2 cells, representing significant progress, especially for MiaPaca‐2 cells, which have been challenging to form spheroids [27, 40].

Following the establishment of PDAC spheroids, we aimed to elucidate the role of AQP3 and AQP5 on PDAC development through the use of AQP inhibitors and knockdown of AQP3 or AQP5. Given the lack of reported AQP5 inhibitors, our initial approach focused exclusively on using AQP3 inhibitors such as Auphen and DFP00173, with reported anticancer properties [41, 42]. It is worth noting that these compounds have been extensively validated as potent AQP3 blockers across multiple cancer cell models [18, 41, 42, 43, 44, 45], including PDAC. In fact, Auphen has previously been tested in BxPC‐3 cells where it significantly reduced AQP3 permeability, showing an effect comparable to AQP3 silencing [18]. Moreover, the impact of AQP3 and AQP5 knockdown on water, glycerol, and H_2_O_2_ permeability in BxPC‐3 cells has been previously characterized [18, 19].

Prior to testing these compounds on PDAC spheroids, we assessed their cytotoxicity in BxPC‐3 and MiaPaca‐2 2D cell cultures to determine a concentration that targeted AQP3 activity without inducing cytotoxic effects. Although Auphen and DFP00173 exhibit similar IC_50_ values in red blood cells for AQP3 inhibition (0.8 μm for Auphen [32] and 0.2 μm for DFP00173 [38]), we still chose to test the maximum nontoxic concentration for both compounds, as their efficacy in cancer cells may differ significantly due to differences in cell membrane composition including lipid [46] and protein profiles [47], which could potentially influence the inhibitory effect of these compounds toward AQP3 activity.

Following treatment of BxPC‐3 and MiaPaca‐2 spheroids with Auphen and DFP00173, a decrease in spheroids' diameter and area was observed, while circularity was only diminished in treated MiaPaca‐2 spheroids. By Day 7, DFP00173 exerted a more prominent effect on the morphology of BxPC‐3 and MiaPaca‐2 spheroids compared to Auphen, except for MiaPaca‐2 spheroids' circularity, which was more significantly affected by Auphen. The lack of differences in BxPC‐3 spheroids' circularity upon treatment with AQP3 inhibitors is likely attributed to their high compactness; however, cell dissociation from the spheroids was observable. In MiaPaca‐2 spheroids, which are less compact, differences in circularity were more noticeable, as spheroids treated with Auphen or DFP00173 exhibited poorly defined borders with distinguishable cells. Moreover, both compounds significantly inhibited BxPC‐3 and MiaPaca‐2 spheroids' proliferation, as evidenced by the decreased viability along with reduced spheroids' diameter and area.

Even though different concentrations were used for Auphen and DFP00173, both AQP3 inhibitors similarly impaired PDAC spheroids' growth, probably via AQP3 inhibition. In fact, AQP3 has been extensively associated with promoting cancer cell proliferation in PDAC, as demonstrated in biopsies showing a correlation between AQP3 and AQP5 overexpression and increased expression of the proliferation marker Ki‐67 [17], and in PDAC cell lines, where AQP3 was found to enhance PDAC proliferation via the mTOR signaling pathway [15].

The implication of AQP3 in cancer proliferation extends beyond PDAC, as its role in tumor growth has been linked with other types of cancer including skin cancer [7, 43, 48], breast cancer [49], hepatocellular carcinoma [50], squamous cell carcinoma, and lung cancer [51]. In addition, Auphen and DFP00173 have demonstrated their anticancer activities through AQP3 inhibition. Auphen was shown to diminish cell proliferation in AQP3‐expressing cells [52] and in an *in vivo* model of hepatocellular carcinoma [41] by targeting AQP3. Moreover, Auphen was found to inhibit AQP3 peroxiporin activity, impairing melanoma cell proliferation, migration, and adhesion [43], while DFP00173 effectively suppressed multiple myeloma growth through AQP3 blockage [42]. These findings further support the observed inhibition of PDAC spheroids' growth by the AQP3 inhibitors Auphen and DFP00173, highlighting the important contribution of AQP3 in PDAC development.

After individually silencing AQP3 and AQP5 in BxPC‐3 cells through the CRISPR‐Cas9 technique to further elucidate the implications of these AQPs in PDAC progression, we validated the AQP3 and AQP5 knockdown and generated 3D cell cultures for control, AQP3 KD, and AQP5 KD cells. Both AQP3 and AQP5 KD spheroids demonstrated a decreased diameter and area, with AQP3 KD spheroids showing a more significant reduction, while circularity was unchanged. Spheroids' viability was significantly impaired in AQP3 KD and AQP5 KD spheroids on Days 4 and 7. On Day 4, impaired cell viability without visible alterations in spheroids' morphology suggested increased cell death and potential necrotic core formation, while on Day 7, reduced spheroids' diameter and area, along with cell dissociation, indicated impaired spheroids' growth and greater cell death.

A similar trend was observed between BxPC‐3 AQP3 KD and BxPC‐3 spheroids treated with Auphen or DFP00173, exhibiting decreased spheroids' diameter and area without affecting circularity. In both conditions, spheroids presented cell dissociation and impaired viability, with a more pronounced decrease for AQP3 KD spheroids on Day 4. Our findings demonstrate that Auphen and DFP00173 selectively and potently inhibit AQP3, resulting in reduced PDAC spheroids' proliferation comparable to that observed in AQP3 KD models, thereby reinforcing the critical contribution of AQP3 to PDAC progression.

AQP5 KD spheroids demonstrated a similar impact on PDAC spheroids' growth as AQP3 KD spheroids, which aligns with the reported implication of AQP5 on cancer cell proliferation. AQP5 was found to promote tumor growth in colon cancer via Ras signaling, probably mediated by AQP5 phosphorylation [53, 54], and in lung cancer through the upregulation of the proliferation marker c‐myc and EGFR/extracellular signal‐regulated kinase (ERK)/p38 mitogen activated protein kinase (MAPK) signaling pathway [55]. Additionally, AQP5 overexpression has been correlated with increased Ki‐67 levels in PDAC biopsies [17], and was associated with breast cancer [49] and hepatocellular carcinoma [50] progression. Thus, AQP3 and AQP5 are key regulators of PDAC proliferation, potentially linked to their transceptor activity, modulating cancer‐related signaling pathways, and interacting with cytoskeleton elements to drive tumorigenesis and metastasis [56, 57, 58, 59].

Progress has been made to develop 3D cell culture models to better understand the importance of AQPs in cancer. However, to the best of our knowledge, this is the first study to report the involvement of AQP3 and AQP5 in PDAC spheroids' growth. Interestingly, the role of AQP3 and AQP5 in breast cancer 3D cell models was recently investigated, with AQP5 overexpression reducing spheroids' size and circularity [60], whereas AQP3 overexpression had no significant effect on spheroids' morphology [61]. Moreover, AQP3 and AQP5 overexpression in breast cancer spheroids was correlated with different sensitivity to conventional chemotherapy drugs [62]. In addition, overexpression of AQP5 was found to enhance the size, cohesiveness, and invasive capacity of breast cancer spheroids, highlighting the pivotal role of AQP5 in promoting tumor progression and invasiveness [63].

Overall, our study demonstrates for the first time the relevance of AQP3 and AQP5 on PDAC spheroids' development. The observed changes in PDAC spheroids' morphology and growth induced by either gene silencing or AQP3 inhibition suggest that PDAC progression is AQP3 and AQP5 dependent and highlight both AQPs as promising therapeutic targets in PDAC.

## Conclusions

Through diverse experimental approaches, this study reveals the involvement of AQP3 and AQP5 in the development and proliferation of PDAC 3D cell cultures, providing insights that may reflect their roles in *in vivo* models. Further investigation into the mechanisms underlying their contribution to PDAC progression—potentially linked to their transceptor functions—is paramount.

## Conflicts of interest

The authors declare no conflicts of interest.

## Author contributions

GS and FM were involved in conceptualization. CP and DME performed the experiments and formal analysis. CP prepared the original draft. CP, DME, GS, and FM performed draft reviewing and editing. GS and FM were involved in resources. All authors have read and approved the final version of this manuscript.

## Supporting information


**Fig. S1.** Original Western Blot images showing (A) AQP3, (B) AQP5 and (C) α‐tubulin protein expression in BxPC‐3 and MiaPaca‐2 cells.
**Fig. S2**. Original Western Blot images showing (A) AQP3, (B) AQP5 and (C) α‐tubulin protein expression in BxPC‐3 cells control and knockdown for AQP3 (AQP3 KD) or AQP5 (AQP5 KD).
**Fig. S3**. Representative microscope images of BxPC‐3 control, AQP3 KD and AQP5 KD spheroids on day 3, 4 and 7.

## Data Availability

The data that support the findings of this study are available in Figs 1, 2, 3, 4, 5, 6 of this study and in the Figs S1–S3.
