# Supplementary material for: Aquaporin‐3 and aquaporin‐5 impact the development of pancreatic ductal adenocarcinoma spheroids
Source: FEBS Open Bio. 2026 May 17:10.1002/2211-5463.70270. Online ahead of print. doi: 10.1002/2211-5463.70270 (PMC13398349; doi:10.1002/2211-5463.70270)
Supplement: Supplementary file 1 — Fig. S1. Original Western Blot images showing (A) AQP3, (B) AQP5 and (C) α‐tubulin protein expression in BxPC‐3 and MiaPaca‐2 cells. Fig. S2. Original Western Blot images showing (A) AQP3, (B) AQP5 and (C) α‐tubulin protein expression in BxPC‐3 cells control and knockdown for AQP3 (AQP3 KD) or AQP5 (AQP5 KD). Fig. S3. Representative microscope images of BxPC‐3 control, AQP3 KD and AQP5 KD spheroids on day 3, 4 and 7. [file FEB4-9999-0-s001.pdf]

Aquaporin-3 and Aquaporin-5 Impact the Development of Pancreatic Ductal Adenocarcinoma Spheroids

Catarina Pimpão[1,2], Diogo M. Engrácia[3], Graça Soveral[1,2*], Filipa Mendes[3,4*]

[1] Research Institute for Medicines (iMed.ULisboa), Faculty of Pharmacy, Universidade de Lisboa, 1649-003 Lisboa, Portugal
[2] Department of Pharmaceutical Sciences and Medicines, Faculty of Pharmacy, Universidade de Lisboa, 1649-003 Lisboa, Portugal
[3] C2TN - Centro de Ciências e Tecnologias Nucleares, Instituto Superior Técnico, Universidade de Lisboa, Lisboa, Portugal.
[4] Departamento de Engenharia e Ciências Nucleares, Instituto Superior Técnico, Universidade de Lisboa, Lisboa, Portugal

SUPPLEMENTAL FIGURES

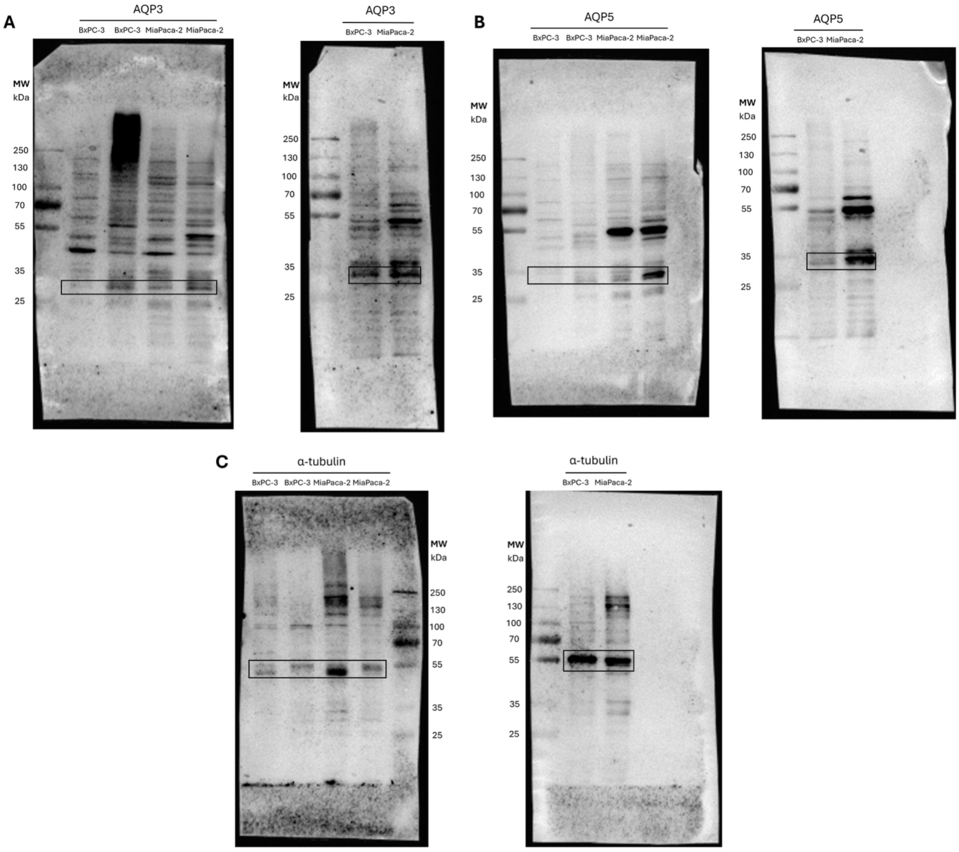

Figure S1. Original Western Blot images showing (A) AQP3, (B) AQP5 and (C) α-tubulin protein expression in BxPC-3 and MiaPaca-2 cells. AQP3 and AQP5 expression levels were normalized to α-tubulin. Molecular weight markers (kDa) are indicated to evaluate the size of the detected bands.

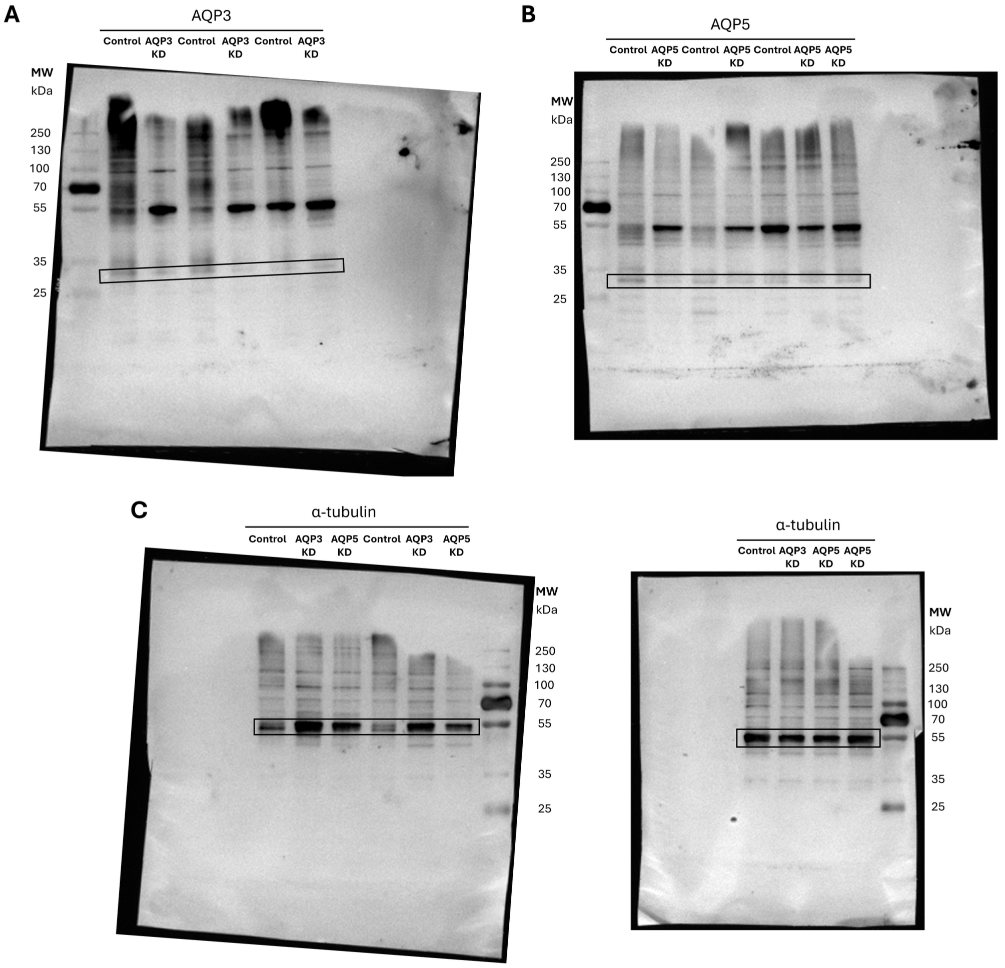

Figure S2. Original Western Blot images showing (A) AQP3, (B) AQP5 and (C) α-tubulin protein expression in BxPC-3 cells control and knockdown for AQP3 (AQP3 KD) or AQP5 (AQP5 KD). AQP3 and AQP5 expression levels were normalized to α-tubulin. Molecular weight markers (kDa) are indicated to evaluate the size of the detected bands.

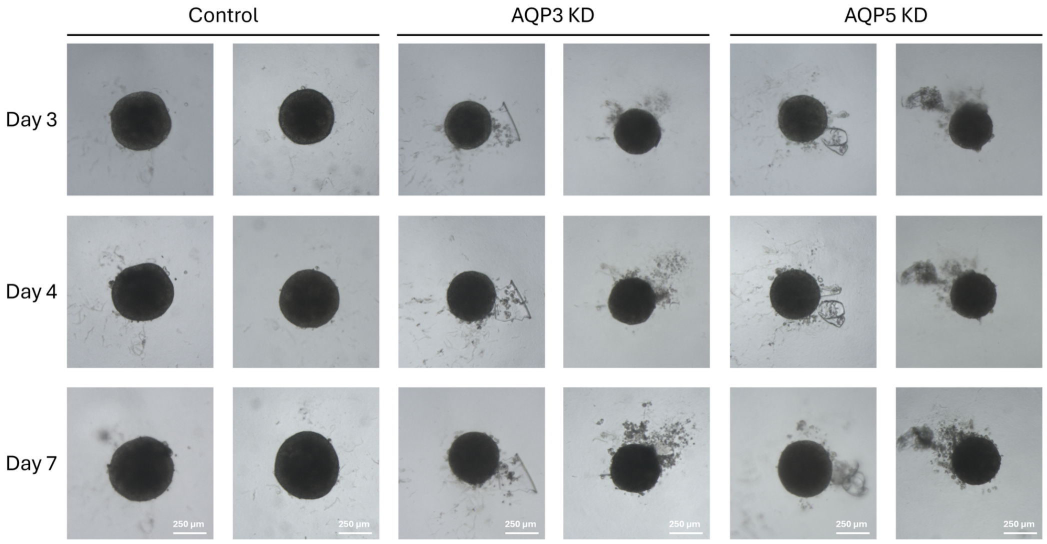

Figure S3. Representative microscope images of BxPC-3 control, AQP3 KD and AQP5 KD spheroids on day 3, 4 and 7. Scale bar: 250 µm.
